# Management of open abdomen complication after laparotomy in an ovarian cancer patient with intraperitoneal metastasis

**DOI:** 10.1093/jscr/rjae482

**Published:** 2024-08-06

**Authors:** Jae Hoon Jeong, Jae Hyun Lee, Chongsoo Park

**Affiliations:** Department of Plastic and Reconstructive Surgery, Seoul National University College of Medicine, Seoul National University Bundang Hospital, 82, Gumi-ro 173 Beon-gil, Bundang-gu, Seongnam-si, Gyeonggi-do 13620, Republic of Korea; Department of Plastic and Reconstructive Surgery, Inje University College of Medicine, Inje University Busan Paik Hospital, 75 Bokji-ro, Busanjin-gu, Busan 47392, Republic of Korea; Department of Plastic and Reconstructive Surgery, Inje University College of Medicine, Inje University Busan Paik Hospital, 75 Bokji-ro, Busanjin-gu, Busan 47392, Republic of Korea

**Keywords:** open abdomen, ovarian cancer, bile leakage, VAC dressing

## Abstract

This case report highlights the management of complications from an open abdomen following surgery for ovarian mucinous adenocarcinoma, a rare subtype of ovarian cancer. A 63-year-old female underwent extensive surgery, including single-port laparoscopic total bilateral salpingo-oophorectomy, right hemicolectomy, small bowel resection, cholecystectomy, and jejunostomy. Postoperatively, she experienced bile leakage, leading to significant skin and fascial damage and an abdominal skin defect. Early detection and multidisciplinary management were crucial. Treatment involved vacuum-assisted closure dressing, repeated debridement, and closure of the open abdomen with a local flap. This case emphasizes the complexities of managing ovarian mucinous adenocarcinoma and the critical role of a multidisciplinary approach in treating postoperative complications, underscoring the importance of vigilant postoperative care and timely intervention.

## Introduction

The concept of an ‘open abdomen (OA)’ refers to a surgical condition where the abdominal wall is left open, typically following extensive surgery or trauma [1]. This approach is used in various critical scenarios, including trauma patients with hypotension, acidosis, hypothermia, and coagulopathy, indicating the need for an abbreviated laparotomy. It is also applied in cases of abdominal compartment syndrome, severe peritonitis, vascular emergencies, and severe acute pancreatitis when conservative management fails [2]. Careful consideration of factors like infection control, fluid management, and the timing of eventual abdominal closure is required when managing an open abdomen [1, 3].

## Case report

**Figure 1:**
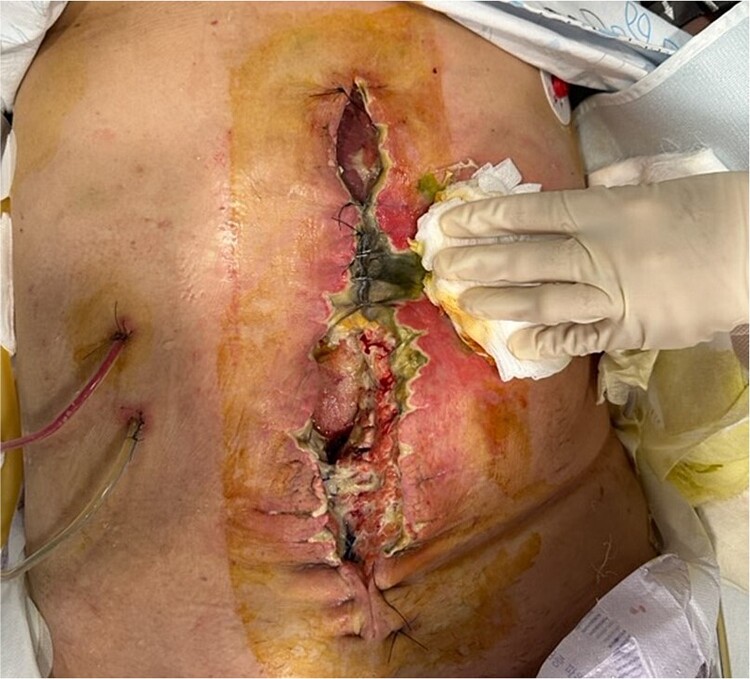
Complication of an open abdomen on 7 days after extensive surgery for ovarian cancer.

A 63-year-old female with ovarian mucinous adenocarcinoma underwent extensive surgery, including single-port laparoscopic total bilateral salpingo-oophorectomy, right hemicolectomy, small bowel resection, cholecystectomy, and jejunostomy, aiming for complete tumor resection. Seven days postoperatively, she developed bile leakage, causing significant damage to the abdominal wall and dehiscence of the midline surgical site (Fig. 1).

**Figure 2:**
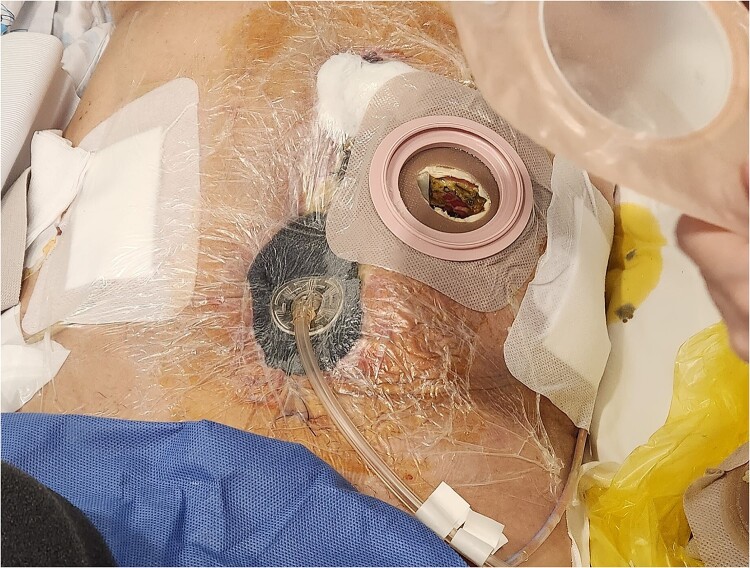
VAC dressing was applied to the open abdominal wound for 26 days.

**Figure 3:**
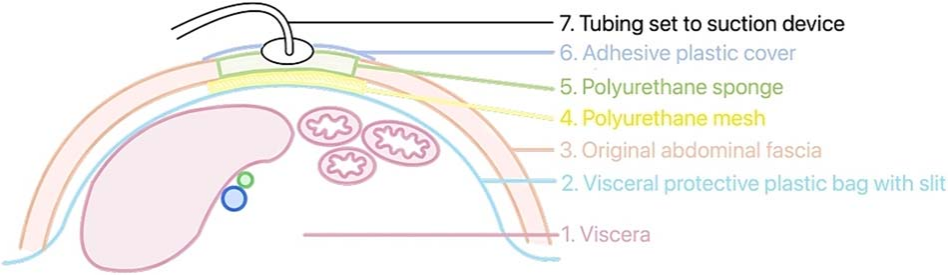
Schematic diagram of VAC system application on a patient.

**Figure 4:**
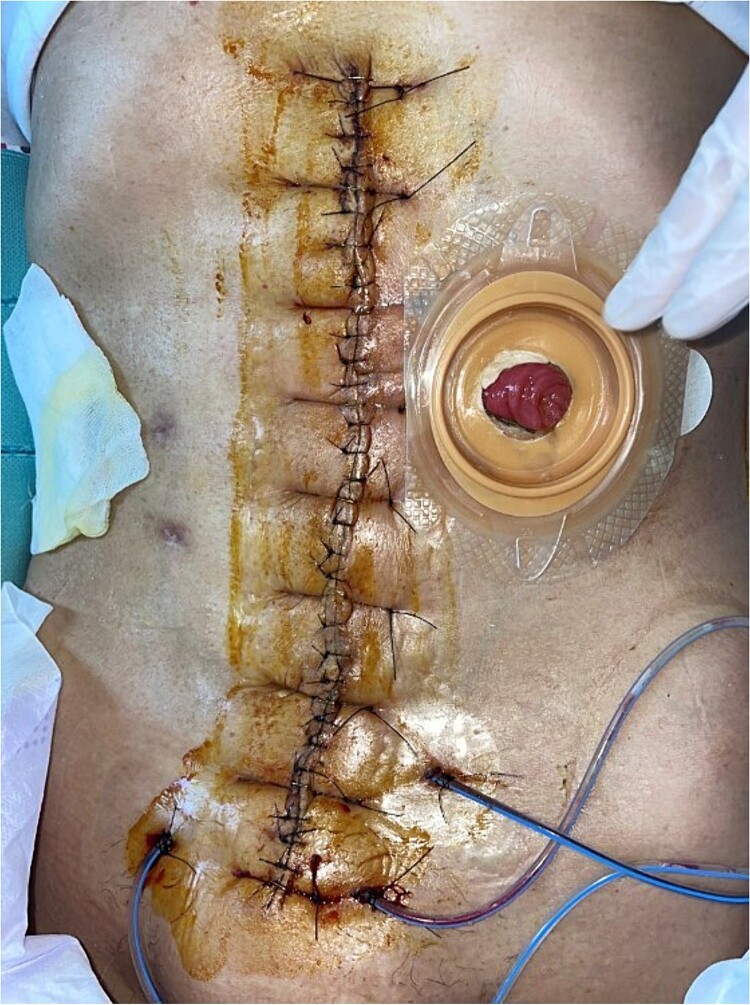
Repeated debridement and primary closure were performed on 1 month postoperatively.

In response, the VAC technique was implemented for 4 weeks, which was essential in managing the open wound and facilitating healing (Fig. 2). When applying VAC, using an organ transport bag as a visceral protective layer is important. This plastic bag should be unfolded into a single layer and slit boldly to facilitate the pressure between the intra-abdominal visceral contents and the VAC content (Fig. 3). Four weeks postoperatively, the VAC was removed, and the wound was successfully closed, marking significant progress in the patient’s recovery (Fig. 4).

**Figure 5:**
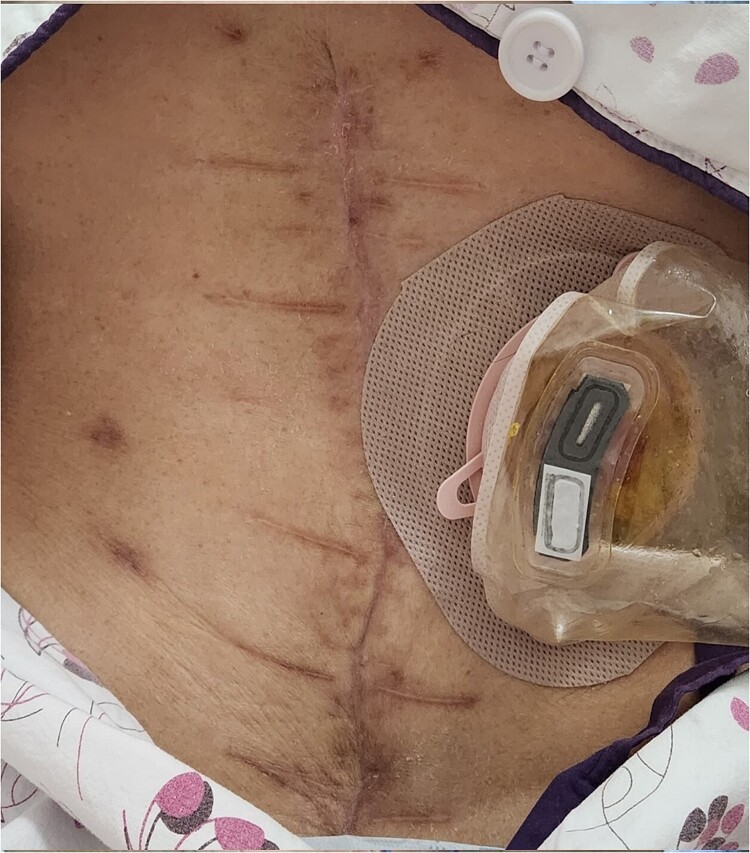
Wound healing was achieved on 2 months postoperatively.

Complete wound healing was documented 2 months after surgery, demonstrating the resilience of both the patient and the medical team in overcoming postoperative challenges (Fig. 5). The bile leakage led to severe skin and fascial damage, resulting in a significant abdominal defect and open abdomen condition, highlighting the risks associated with complex surgical interventions and the unpredictable nature of postoperative recovery.

## Discussion

The management of severe abdominal complications following surgery for metastatic ovarian cancer presents unique challenges. Ovarian mucinous adenocarcinoma, a particularly complex subtype, often requires extensive surgical intervention to remove the tumor and improve outcomes [4, 5]. This case study presents a 63-year-old female patient who underwent multiple surgical interventions, including laparoscopic salpingo-oophorectomy and open abdominal surgery. These procedures were complicated by bile leakage, leading to subsequent open abdomen complications [6]. Management of these complications required a comprehensive, multidisciplinary approach [7], highlighting the importance of early detection, innovative treatment strategies, such as vacuum-assisted closure (VAC), and surgical closure with a local flap, and the crucial role of collaborative care in managing advanced ovarian cancer.

A multidisciplinary approach was crucial in addressing the complications encountered in this case [7]. The collaborative efforts of gynecologic surgeons, gastroenterologists, wound care experts, and nutritionists played a pivotal role in devising a holistic management plan involving VAC dressings, repeated debridement, and closure of the open abdomen with a local flap. This teamwork underscores the significance of a collective approach in tackling complex medical challenges.

Over time, the concept of the OA has evolved as a crucial component of both emergency and planned abdominal surgeries [8]. Recognized as a lifesaving measure, it also carries risks, such as infection, fluid loss, and the challenge of definitive closure. Managing an open abdomen, particularly in oncological surgery, requires a balance of prompt surgical intervention, careful wound care, and continuous monitoring for complications [9]. This case accentuates the need for understanding these dynamics, emphasizing an evidence-based approach in abdominal surgery decision-making.

Advancements in surgical techniques and wound care offer promising improvements in outcomes for patients with complex abdominal complications. The adoption of VAC therapy and the strategic use of local flaps for wound closure are notable advancements. These methods promote effective wound healing, reduce infection risks, and enhance overall recovery. This case emphasizes the importance of incorporating innovative treatment approaches into standard care protocols for open abdomen management and advocates for ongoing advancements in surgical care.

Ovarian cancer, especially the mucinous adenocarcinoma subtype, poses unique surgical challenges due to its aggressive nature and potential for extensive intraperitoneal metastasis [10]. Comprehensive surgical interventions for tumor removal can lead to significant complications. These challenges highlight the need for a carefully crafted surgical plan, anticipation of potential complications, and readiness to implement alternative strategies. This situation offers an opportunity to refine surgical and postoperative care techniques to minimize complications and improve patient outcomes.

Managing OA complications in ovarian cancer patients highlights the intricacies and challenges inherent in contemporary surgical oncology. Insights from this case enrich our understanding of essential aspects in handling such complications, underscoring the need for innovation, teamwork, and patient-centered care. Applying lessons learned from complex cases like this one is crucial to refining approaches, improving patient outcomes, and navigating the complex terrain of oncological surgery with increased accuracy and compassion.

## Data Availability

No new data were generated or analyzed in support of this case.
